# The residual risk of inflammation and remnant cholesterol in acute coronary syndrome patients on statin treatment undergoing percutaneous coronary intervention

**DOI:** 10.1186/s12944-024-02156-3

**Published:** 2024-06-07

**Authors:** Jia Liao, Miaohan Qiu, Xiaolin Su, Zizhao Qi, Ying Xu, Haiwei Liu, Kai Xu, Xiaozeng Wang, Jing Li, Yi Li, Yaling Han

**Affiliations:** 1 State Key Laboratory of Frigid Zone Cardiovascular Disease, Department of Cardiology, General Hospital of Northern Theater Command, Shenyang, 110016 China; 2 Department of Cardiology, Union Hospital, Tongji Medical College, Huazhong University of Science and Technology, Wuhan, 430022 China

**Keywords:** High-sensitivity C-reactive protein, Remnant cholesterol, Residual risk, Acute coronary syndrome, Ischemic events

## Abstract

**Background:**

Residual risk assessment for acute coronary syndrome (ACS) patients after sufficient medical management remains challenging. The usefulness of measuring high-sensitivity C-reactive protein (hsCRP) and remnant cholesterol (RC) in assessing the level of residual inflammation risk (RIR) and residual cholesterol risk (RCR) for risk stratification in these patients needs to be evaluated.

**Methods:**

Patients admitted for ACS on statin treatment who underwent percutaneous coronary intervention (PCI) between March 2016 and March 2019 were enrolled in the analysis. The included patients were stratified based on the levels of hsCRP and RC during hospitalization. The primary outcome was ischemic events at 12 months, defined as a composite of cardiac death, myocardial infarction, or stroke. The secondary outcomes included 12-month all-cause death and cardiac death.

**Results:**

Among the 5778 patients, the median hsCRP concentration was 2.60 mg/L and the median RC concentration was 24.98 mg/dL. The RIR was significantly associated with ischemic events (highest hsCRP tertile vs. lowest hsCRP tertile, adjusted hazard ratio [aHR]: 1.52, 95% confidence interval [CI]: 1.01–2.30, *P* = 0.046), cardiac death (aHR: 1.77, 95% CI:1.02–3.07, *P* = 0.0418) and all-cause death (aHR: 2.00, 95% CI: 1.24–3.24, *P* = 0.0048). The RCR was also significantly associated with these outcomes, with corresponding values for the highest tertile of RC were 1.81 (1.21–2.73, *P* = 0.0043), 2.76 (1.57–4.86, *P* = 0.0004), and 1.72 (1.09–2.73, *P* = 0.0208), respectively. The risks of ischemic events (aHR: 2.80, 95% CI: 1.75–4.49, *P* < 0.0001), cardiac death (aHR: 4.10, 95% CI: 2.18–7.70, *P* < 0.0001), and all-cause death (aHR: 3.00, 95% CI, 1.73–5.19, *P* < 0.0001) were significantly greater in patients with both RIR and RCR (highest hsCRP and RC tertile) than in patients with neither RIR nor RCR (lowest hsCRP and RC tertile). Notably, the RIR and RCR was associated with an increased risk of ischemic events especially in patients with adequate low-density lipoprotein cholesterol (LDL-C) control (LDL-C < 70 mg/dl) (*P*_*interaction*_=0.04). Furthermore, the RIR and RCR provide more accurate evaluations of risk in addition to the GRACE score in these patients [areas under the curve (AUC) for ischemic events: 0.64 vs. 0.66, *P* = 0.003].

**Conclusion:**

Among ACS patients receiving contemporary statin treatment who underwent PCI, high risks of both residual inflammation and cholesterol, as assessed by hsCRP and RC, were strongly associated with increased risks of ischemic events, cardiac death, and all-cause death.

**Supplementary Information:**

The online version contains supplementary material available at 10.1186/s12944-024-02156-3.

## Introduction

Annually, an estimated 7 million individuals worldwide are thought to receive an acute coronary syndrome (ACS) diagnosis, which imposes a significant disease burden [1]. Although substantial advancements have been made in the diagnosis and treatment of ACS, it remains one of the primary causes of global mortality [2]. Previous studies have revealed that even after the administration of current evidence-based therapies such as revascularization and intensive statin therapy, ACS patients still carry significant residual risks for cardiovascular death and thrombotic complications [3]. Therefore, it is essential to identify the determinants of residual risk factors and provide individualized treatment to improve their prognosis.

Typically, the residual cardiovascular risk mainly consists of two components: residual inflammatory risk (RIR) and residual cholesterol risk (RCR) [4, 5]. The RIR refers to the persistent subclinical vascular inflammation, which is a significant factor in recurrent atherothrombotic events in discharged patients and can be assessed by measuring the levels of several signaling molecules, such as high-sensitivity C-reactive protein (hsCRP) and interleukin-6 (IL-6) [6–9]. RCR refers to the remaining elevated levels of atherogenic lipoproteins despite the implementation of lipid-lowering therapies and is mostly defined as the low-density lipoprotein cholesterol (LDL-C) level [7, 10, 11]. A recently published collaborative study indicated that regardless of LDL-C level, individuals with elevated hsCRP were at significant cardiovascular risk [7]. Therefore, it may be crucial to consider utilizing a new residual risk marker associated with atherogenic dyslipidemia in conjunction with hsCRP to accurately determine residual cardiovascular risk. Remnant cholesterol (RC) is the amount of cholesterol carried by triglyceride-rich lipoproteins and has been established as a causative factor for an elevated risk of cardiovascular diseases [12, 13]. Furthermore, it was suggested that RC plays a significant role in addressing the residual risk of cardiovascular events beyond the impact of LDL-C in primary as well as secondary preventive populations [14–18]. However, the synergistic potential of combining RC as a marker of RCR with hsCRP as an indicator of RIR for enhancing risk stratification has not yet been explored.

Thus, this study hypothesize that dual residual risk elevation as assessed by hsCRP and RC may confer a greater risk of ischemic events among ACS patients receiving contemporary statin treatment who underwent percutaneous coronary intervention (PCI). The primary goal of this investigation was to assess the independent and combined prognostic value of hsCRP and RC in a large and contemporary cohort of real-world patients.

## Method

### Study design

The study cohort was derived from a prospective, real-world, single-center registry at the General Hospital of Northern Theater Command in Shenyang, China, which recruited consecutive patients receiving PCI for coronary artery disease between March 2016 and March 2019 [19, 20]. The inclusion criteria for the study were as follows: (1) patients aged 18 years or older, (2) patients diagnosed with ACS who underwent PCI, and (3) patients who had been prescribed statin therapy before PCI. Individuals without comprehensive information on lipid characteristics, such as total cholesterol (TC), high-density lipoprotein cholesterol (HDL-C), and triglycerides, as well as inflammatory biomarkers, were excluded. The institutional ethics committee of the General Hospital of Northern Theater Command accepted this study and waived the need for formal informed consent. The study also met the standards of the Declaration of Helsinki.

### Laboratory analysis and data collection

Blood samples were collected from each patient during hospitalization, subsequent to the procedural intervention. All indicators were measured using standard hospital assays. The level of hsCRP was measured using a Cobas c 501 analyzer (Roche Diagnostics, Mannheim, Germany), and the levels of lipid traits were analyzed using a Beckman Coulter AU5800 (Beckman Coulter Inc., Brea, CA). According to the dyslipidemia guidelines, RC was computed by the following equation: TC - LDL-C - HDL-C [21, 22]. The enrolled patients were categorized into three tertiles (lowest, middle, and highest) based on hsCRP and RC concentrations.

A standard web-based data collection system (CV-NET, Crealife Technology) was used to collect patients’ demographic and clinical characteristics, including age, sex, medical history, ACS type, laboratory findings, angiographic and procedural characteristics, and medication treatment.

### Outcomes and follow-up

The primary outcome was ischemic events at 12 months, defined as a composite of cardiac death, myocardial infarction (MI), or stroke. The secondary outcomes included 12-month all-cause death and cardiac death. Clinical follow-ups were routinely conducted at 3, 6, 9, and 12 months after the procedure or at unscheduled readmission by qualified research nurses or doctors via phone or outpatient visits. Every clinical incident was reviewed by a clinical events committee.

### Statistical analysis

Continuous variables are reported as the mean ± standard deviation (SD) or median (Q1-Q3 quartiles) as appropriate and were compared using analysis of variance or the Kruskal‒Wallis test. Categorical variables are presented as numbers (percentages) and were compared using the χ2 test or Fisher’s exact test. Time-to-event outcomes were analyzed by the Kaplan‒Meier method and compared by the log-rank test. Cox proportional hazards models were used to estimate the hazard ratio (HR) and 95% confidence interval (CI) for each outcome among the groups. To address potential confounding factors, multivariable regression models were employed, adjusting for covariates such as age, sex, hypertension, diabetes, previous MI, previous PCI, previous stroke, smoking, type of ACS, anemia, estimated glomerular filtration rate, arterial access, coronary arteries treated, and number of stents.

The patients were classified into four groups to evaluate the combined prognostic effect of hsCRP and RC: patients in hsCRP tertiles 1 and 2 and RC tertiles 1 and 2 were defined as having no residual risk; patients in hsCRP tertiles 1 and 2 and RC tertile 3 were defined as having RCR; patients in hsCRP tertile 3 and RC tertiles 1 and 2 were defined as having RIR; and patients in hsCRP tertile 3 and RC tertile 3 were defined as having residual cholesterol and residual inflammation risk (RCIR). The subgroup analyses were further stratified by age (< 65 years or ≥ 65 years), sex (male or female), presence of diabetes (yes or no), and LDL-C level (< 70 mg/dL or ≥ 70 mg/dL).

The nonlinear associations between residual risk markers (hsCRP and RC) and 12-month ischemic events, cardiac death, and all-cause death were evaluated using a restricted cubic spline (RCS). Additionally, we performed receiver operating characteristic (ROC) curve analysis and computed the areas under the curve (AUC) to evaluate whether the addition of hsCRP and RC to the global registry of acute coronary events (GRACE) score could improve the ability to predict the outcome events [23]. The ROC curve comparisons were performed in accordance with the methods of DeLong et al [24]. Unless otherwise noted, a two-sided *P* value less than 0.05 indicated statistical significance. The statistical analysis was conducted using SAS software version 9.4 (SAS Institute, Cary, NC, USA).

## Results

### Baseline characteristics

A total of 5778 patients were included in the study. The median (interquartile range) hsCRP level was 2.60 (1.10, 7.40) mg/L, and the median (interquartile range) RC level was 24.98 (17.71, 34.90) mg/dL. As Table 1 illustrates, patients with elevated hsCRP levels were older and presented more frequently with STEMI. Additionally, these patients had a greater incidence of hypertension, diabetes, anemia, and active smoking; a lower incidence of previous MI and PCI; greater levels of TC and LDL-C; and lower levels of HDL-C. Moreover, patients with elevated RC who were younger exhibited a lower incidence of STEMI. A greater proportion of these patients were female, and there was a greater incidence of cardiovascular risk factors such as hypertension, diabetes and active smoking. Regarding procedural characteristics, most factors were balanced, except that the stent length in patients with elevated hsCRP was likely longer and the number of stents in patients with elevated RC was likely greater. Regarding the medications at discharge, almost all patients had undergone antiplatelet therapy with aspirin. Patients with elevated hsCRP and RC were more likely to be prescribed ACEI/ARB and β-blocker.

**Table 1:** Baseline characteristics of individuals by tertiles of high-sensitivity C-reactive protein and remnant cholesterol

	High-sensitivity C-reactive protein			*P* value		Remnant cholesterol			*P* value
	Tertiles 1 (*N* = 2005)	Tertiles 2 (*N* = 1847)	Tertiles 3 (*N* = 1926)			Tertiles 1 (*N* = 1895)	Tertiles 2 (*N* = 1935)	Tertiles 3 (*N* = 1948)	
Age, years	60.37 ± 10.04	60.51 ± 10.85	60.96 ± 11.64	0.0345		63.29 ± 10.41	60.73 ± 10.71	57.89 ± 10.75	< 0.0001
Male	1529(76.26%)	1390(75.26%)	1408(73.10%)	0.0674		1488(78.52%)	1437(74.26%)	1402(71.97%)	< 0.0001
Medical history									
Hypertension	1107(55.21%)	1096(59.34%)	1176(61.12%)	0.0006		1049(55.36%)	1118(57.81%)	1212(62.25%)	< 0.0001
Diabetes	533(26.64%)	573(31.07%)	627(32.67%)	0.0001		456(24.11%)	571(29.57%)	706(36.35%)	< 0.0001
Previous MI	341(17.02%)	279(15.15%)	218(11.37%)	< 0.0001		292(15.45%)	287(14.88%)	259(13.33%)	0.1553
Previous PCI	503(25.09%)	352(19.06%)	272(14.14%)	< 0.0001		388(20.47%)	387(20.02%)	352(18.08%)	0.1365
Previous stroke	246(12.30%)	273(14.81%)	313(16.29%)	0.0016		285(15.07%)	290(15.03%)	257(13.22%)	0.1747
Smoking				< 0.0001					0.0007
Never	849(42.41%)	686(37.22%)	747(38.89%)			772(40.80%)	778(40.29%)	732(37.67%)	
Active	884(44.16%)	925(50.19%)	969(50.44%)			865(45.72%)	905(46.87%)	1008(51.88%)	
Former	269(13.44%)	232(12.59%)	205(10.67%)			255(13.48%)	248(12.84%)	203(10.45%)	
Type of ACS				< 0.0001					< 0.0001
UA	1085(54.11%)	705(38.17%)	478(24.82%)			780(41.16%)	737(38.09%)	751(38.55%)	
NSTEMI	264(13.17%)	355(19.22%)	486(25.23%)			280(14.78%)	394(20.36%)	431(22.13%)	
STEMI	656(32.72%)	787(42.61%)	962(49.95%)			835(44.06%)	804(41.55%)	766(39.32%)	
Anemia	231(11.52%)	261(14.15%)	463(24.04%)	< 0.0001		356(18.80%)	352(18.19%)	247(12.69%)	< 0.0001
eGFR, mL/min per 1.73 m2	93.42 ± 21.80	89.98 ± 24.20	86.04 ± 25.78	< 0.0001		90.22 ± 22.36	88.83 ± 24.39	90.52 ± 25.50	0.0441
Total cholesterol, mg/dL	160.48 ± 43.90	175.40 ± 47.88	175.95 ± 48.29	< 0.0001		153.37 ± 40.67	168.24 ± 42.47	189.13 ± 50.79	< 0.0001
LDL-C, mg/dL	92.77 ± 37.35	104.07 ± 40.02	107.25 ± 38.28	< 0.0001		96.68 ± 36.10	103.82 ± 38.58	103.02 ± 41.75	< 0.0001
HDL-C, mg/dL	41.31 ± 10.01	40.53 ± 10.84	39.02 ± 9.74	< 0.0001		41.62 ± 9.83	39.56 ± 9.17	39.74 ± 11.44	< 0.0001
GRACE risk score	83.62 ± 21.71	85.43 ± 23.47	88.87 ± 25.02	< 0.0001		91.09 ± 22.90	86.06 ± 23.73	80.82 ± 22.78	< 0.0001
Transradial access	1892(94.36%)	1718(93.02%)	1768(91.80%)	0.0065		1766(93.19%)	1804(93.23%)	1808(92.81%)	0.8521
Coronary arteries treated									
LM	88(4.39%)	78(4.22%)	83(4.31%)	0.9684		83(4.38%)	95(4.91%)	71(3.64%)	0.1496
LAD	1049(52.32%)	930(50.35%)	990(51.40%)	0.4748		989(52.19%)	1001(51.73%)	979(50.26%)	0.4545
LCX	416(20.75%)	394(21.33%)	429(22.27%)	0.5021		390(20.58%)	406(20.98%)	443(22.74%)	0.2198
RCA	746(37.21%)	719(38.93%)	716(37.18%)	0.4465		705(37.20%)	723(37.36%)	753(38.66%)	0.5937
Number of stents	1.42 ± 0.79	1.42 ± 0.84	1.46 ± 0.85	0.1899		1.39 ± 0.84	1.44 ± 0.81	1.46 ± 0.82	0.0293
Total length of stents, mm	39.87 ± 22.36	41.15 ± 22.82	42.51 ± 23.59	0.0024		40.23 ± 22.62	41.46 ± 22.96	41.72 ± 23.22	0.1208
Average stent diameters, mm	3.07 ± 0.67	3.06 ± 0.88	3.03 ± 0.59	0.1929		3.06 ± 0.66	3.05 ± 0.86	3.05 ± 0.61	0.9369
Medications at discharge									
Aspirin	1991(99.30%)	1832(99.19%)	1912(99.27%)	0.9137		1877(99.05%)	1920(99.22%)	1938(99.49%)	0.2843
P2Y12 inhibitors				0.1685					0.0016
Clopidogrel	1224(61.17%)	1161(63.24%)	1229(63.98%)			1221(64.71%)	1234(64.07%)	1159(59.59%)	
Ticagrelor	777(38.83%)	675(36.76%)	692(36.02%)			666(35.29%)	692(35.93%)	786(40.41%)	
ACEI/ARB	1299(64.79%)	1280(69.30%)	1344(69.78%)	0.0011		1262(66.60%)	1292(66.77%)	1369(70.28%)	0.0217
β-blockers	1365(68.08%)	1308(70.82%)	1397(72.53%)	0.0085		1278(67.44%)	1369(70.75%)	1423(73.05%)	0.0007
Statin				0.1651					0.0793
Atorvastatin	471/1732 (27.19%)	471/1628 (28.93%)	528/1724 (30.63%)			496/1642 (30.21%)	504/1703 (29.59%)	470/1739 (27.03%)	
Rosuvastatin	1071/1732 (61.84%)	984/1628 (60.44%)	1035/1724 (60.03%)			961/1642 (58.53%)	1034/1703 (60.72%)	1095/1739 (62.97%)	
Others*	190/1732 (10.97%)	173/1628 (10.63%)	161/1724 (9.34%)			185/1642 (11.27%)	165/1703 (9.69%)	174/1739 (10.01%)	

Abbreviations: MI, myocardial infarction; PCI, percutaneous coronary intervention; ACS, acute coronary syndrome; UA, unstable angina; NSTEMI, non-ST-elevation myocardial infarction;

STEMI, ST-elevation myocardial infarction; eGFR, estimated glomerular filtration rate; LDL-C, low-density lipoprotein cholesterol; HDL-C, high-density lipoprotein cholesterol; GRACE, global registry of acute coronary events; LM, left main; LAD, left anterior descending; LCx, left circumflex; RCA, right coronary artery; ACEI, angiotension converting enzyme inhibitors; ARB, angiotensin II receptor blocker

*Other statins include simvastatin, pravastatin, fluvastatin, and pitavastatin

### Individual effects of hsCRP on outcomes

**Fig. 1:**
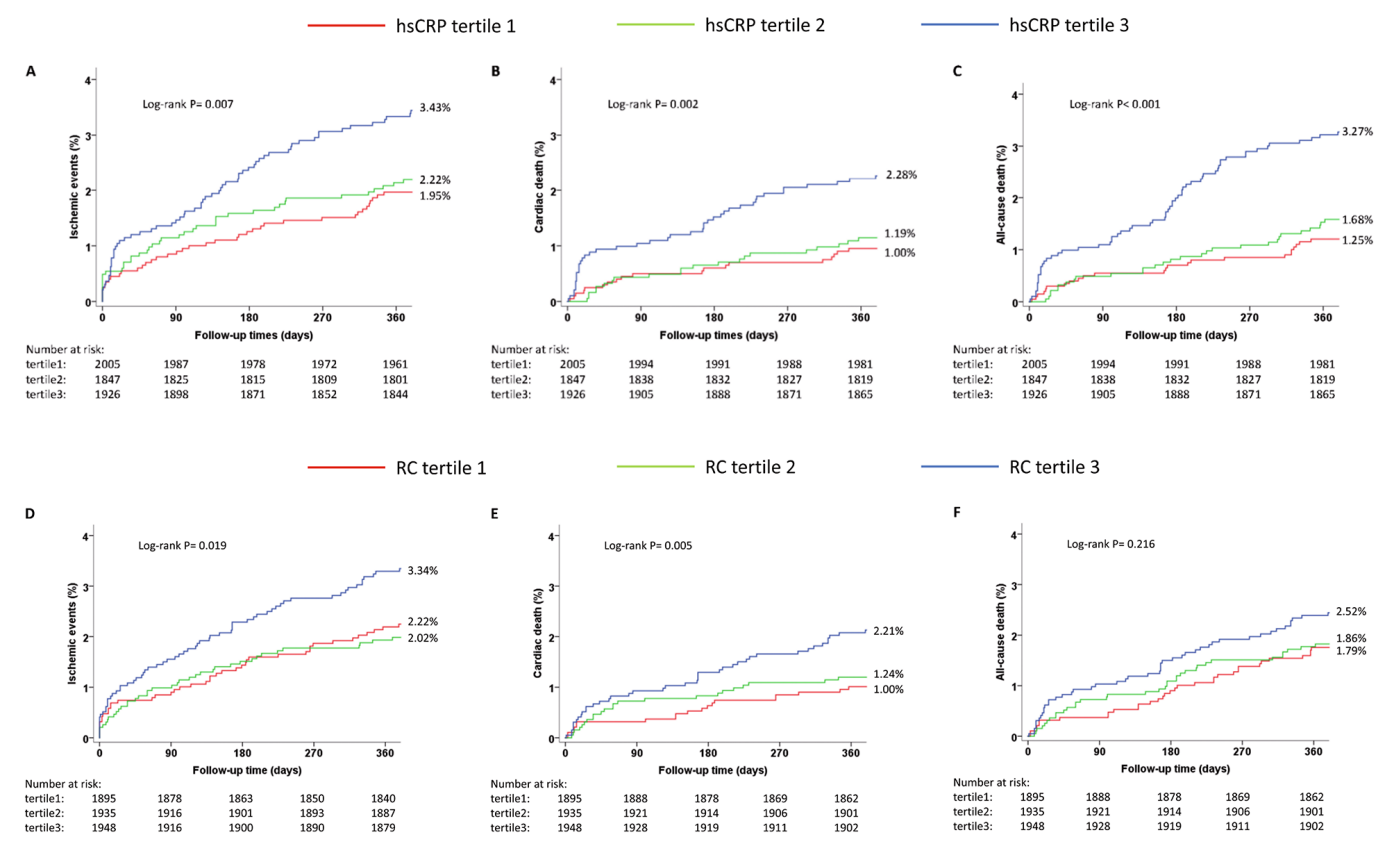
The cumulative Kaplan-Meier analyses according to high-sensitivity C-reactive protein or remnant cholesterol (hsCRP: **A**. ischemic events **B**. cardiac death **C**. all-cause death; RC: **D**. ischemic events **E**. cardiac death **F**. all-cause death)

**Fig. 2:**
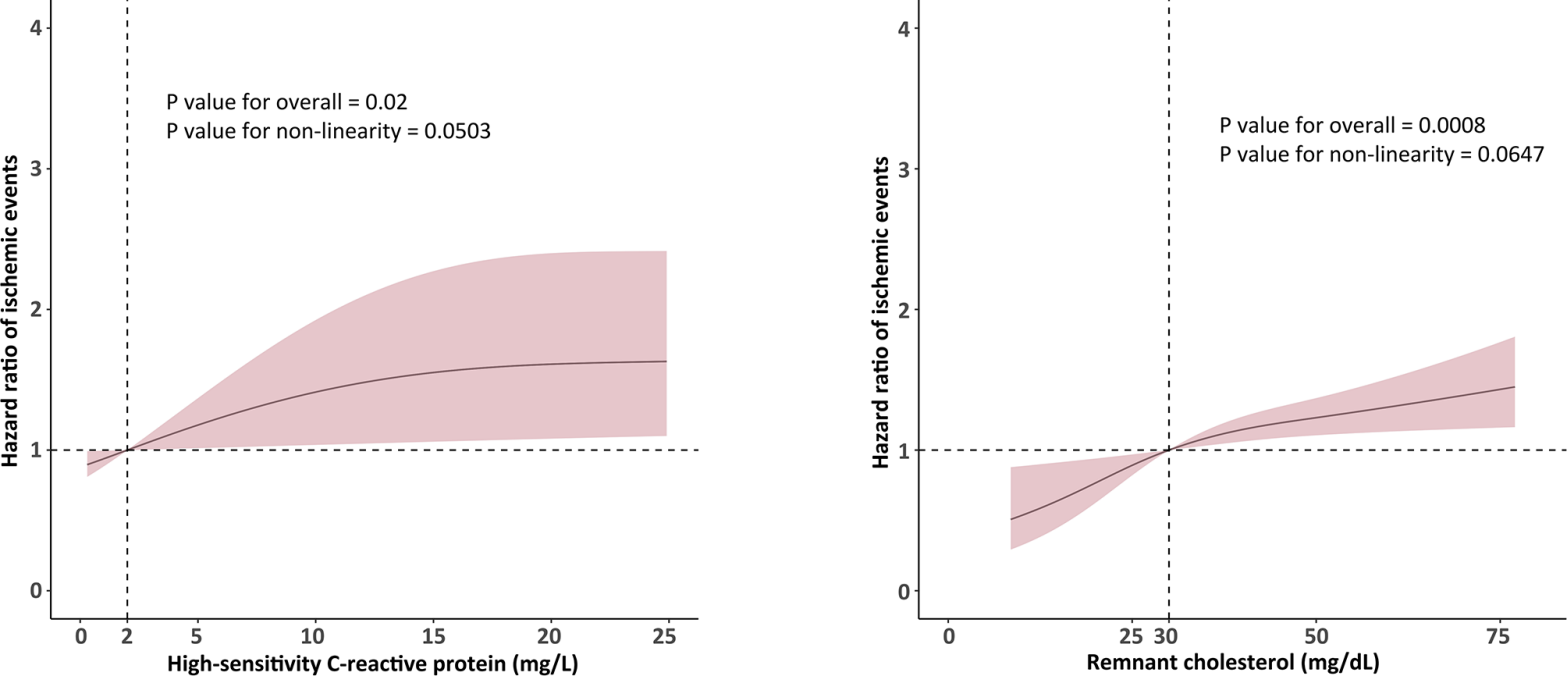
Restricted cubic spline fitting for the association between high-sensitivity C-reactive protein and remnant cholesterol with ischemic events

The primary outcome of 12-month ischemic events occurred in 39 (1.95%), 41 (2.22%), and 66 (3.43%) patients in the lowest, middle, and highest tertiles of hsCRP level group, respectively (log-rank *P* = 0.007). The incidences of 12-month all-cause death and cardiac death were also greater in the highest tertile of hsCRP level group. The Kaplan‒Meier analysis results are shown in Fig. 1-ABC. Compared with patients in tertile 1, the adjusted hazard ratio (aHR) (95% CI) for those in tertile 3 were 1.52 (1.01–2.30) for ischemic events (*P* = 0.046), 1.77 (1.02–3.07) for cardiac death (*P* = 0.0418), and 2.00 (1.24–3.24) for all-cause death (*P* = 0.0048) (Table 2). The adjusted Kaplan‒Meier analysis results are shown in Supplementary 1. When hsCRP was analyzed as a continuous variable, as the concentration per 1 mg/L increase, the risk of ischemic events (aHR: 1.005, *P* = 0.0311), cardiac death (aHR: 1.007, *P* = 0.0043) and all-cause death (aHR: 1.006, *P* = 0.0012) also significantly increased (Table 2). The RCS curves demonstrated a positive correlation between the risk of hsCRP and ischemic events (overall *P* value = 0.02, *P* for nonlinearity = 0.0503) (Fig. 2A). The RCS curves between hsCRP and cardiac death or all-cause death are shown in Supplementary 2–3.

**Table 2:** Cox regression analyses of high-sensitivity C-reactive protein for predicting clinical outcomes

High-sensitivity C-reactive protein	Incidence (%)	Hazard ratio (95%CI)	*P* value	Adjusted hazard ratio (95%CI)	*P* value
**Ischemic events**					
Tertiles 1	1.95% (39/2005)	Reference	-	Reference	-
Tertiles 2	2.22% (41/1847)	1.14 (0.74–1.77)	0.546	1.04 (0.67–1.62)	0.8632
Tertiles 3	3.43% (66/1926)	1.78 (1.20–2.64)	0.0043	1.52 (1.01–2.30)	0.046
hs-CRP per 1 mg/L increase	-	1.006 (1.002–1.010)	0.0018	1.005 (1.000-1.009)	0.0311
**Cardiac death**					
Tertiles 1	1.00% (20/2005)	Reference	-	Reference	-
Tertiles 2	1.19% (22/1847)	1.19 (0.65–2.19)	0.5644	1.03 (0.56–1.90)	0.9264
Tertiles 3	2.28% (44/1926)	2.31 (1.36–3.93)	0.0019	1.77 (1.02–3.07)	0.0418
hs-CRP per 1 mg/L increase	-	1.009 (1.005–1.013)	< 0.0001	1.007 (1.002–1.011)	0.0043
**Death from any cause**					
Tertiles 1	1.25% (25/2005)	Reference	-	Reference	-
Tertiles 2	1.68% (31/1847)	1.35 (0.80–2.28)	0.2675	1.18 (0.70–2.01)	0.5331
Tertiles 3	3.27% (63/1926)	2.65 (1.67–4.22)	< 0.0001	2.00 (1.24–3.24)	0.0048
hs-CRP per 1 mg/L increase	-	1.009 (1.006–1.013)	< 0.0001	1.006 (1.002–1.010)	0.0012

Model adjusted for age, sex, hypertension, diabetes, previous myocardial infarction, previous percutaneous coronary intervention, previous stroke, smoking, type of ACS, anemia, eGFR, arterial access, coronary arteries treated, and number of stents

### Individual effects of RC on outcomes

The primary outcome of 12-month ischemic events occurred in 42 (2.22%), 39 (2.02%), and 65 (3.34%) patients in the lowest, middle, and highest tertiles of RC level group, respectively (log-rank *P* = 0.019). The incidences of 12-month all-cause death and cardiac death were also greater in the highest tertile of RC level group. The Kaplan‒Meier analysis results are shown in Fig. 1-DEF. Compared with patients in tertile 1, the aHR (95% CI) for those in tertile 3 were 1.81 (1.21–2.73) for ischemic events (*P* = 0.0043), 2.76 (1.57–4.86) for cardiac death (*P* = 0.0004), and 1.72 (1.09–2.73) for all-cause death (*P* = 0.0208) (Table 3). The adjusted Kaplan‒Meier analysis results are shown in Supplementary 1. When RC was analyzed as a continuous variable, as the concentration per 10 mg/dL increased, the risk of ischemic events (aHR: 1.08, *P* = 0.0001), cardiac death (aHR: 1.11, *P* < 0.0001) and all-cause death (aHR: 1.10, *P* < 0.0001) also significantly increased (Table 3). The RCS curves demonstrated a positive correlation between the risk of RC and ischemia events (overall *P* value = 0.0008, *P* for nonlinearity = 0.0647) (Fig. 2B). The RCS curves between RC and cardiac death or all-cause death are shown in Supplementary 2–3.

**Table 3:** Cox regression analyses of remnant cholesterol for predicting clinical outcomes

Remnant cholesterol	Incidence (%)	Hazard ratio (95%CI)	*P* value	Adjusted hazard ratio (95%CI)	*P* value
**Ischemic events**					
Tertiles 1	2.22% (42/1895)	Reference	-	Reference	-
Tertiles 2	2.02% (39/1935)	0.91 (0.59–1.41)	0.6669	0.95 (0.61–1.48)	0.8376
Tertiles 3	3.34% (65/1948)	1.51 (1.03–2.23)	0.0364	1.81 (1.21–2.73)	0.0043
RC per 10 mg/dL increase	-	1.07 (1.03–1.12)	0.0012	1.08 (1.04–1.12)	0.0001
**Cardiac death**					
Tertiles 1	1.00% (19/1895)	Reference	-	Reference	-
Tertiles 2	1.24% (24/1935)	1.24 (0.68–2.26)	0.4858	1.29 (0.70–2.38)	0.4062
Tertiles 3	2.21% (43/1948)	2.21 (1.29–3.80)	0.004	2.76 (1.57–4.86)	0.0004
RC per 10 mg/dL increase	-	1.09 (1.05–1.14)	0.0001	1.11 (1.06–1.16)	< 0.0001
**Death from any cause**					
Tertiles 1	1.79% (34/1895)	Reference	-	Reference	-
Tertiles 2	1.86% (36/1935)	1.04 (0.65–1.66)	0.8747	1.05 (0.65–1.69)	0.8354
Tertiles 3	2.52% (49/1948)	1.41 (0.91–2.18)	0.1244	1.72 (1.09–2.73)	0.0208
RC per 10 mg/dL increase	-	1.07 (1.03–1.12)	0.0025	1.10 (1.05–1.15)	< 0.0001

Model adjusted for age, sex, hypertension, diabetes, previous myocardial infarction, previous percutaneous coronary intervention, previous stroke, smoking, type of ACS, anemia, eGFR, arterial access, coronary arteries treated, and number of stents

### Joint effects of hsCRP and RC on outcomes

**Fig. 3:**
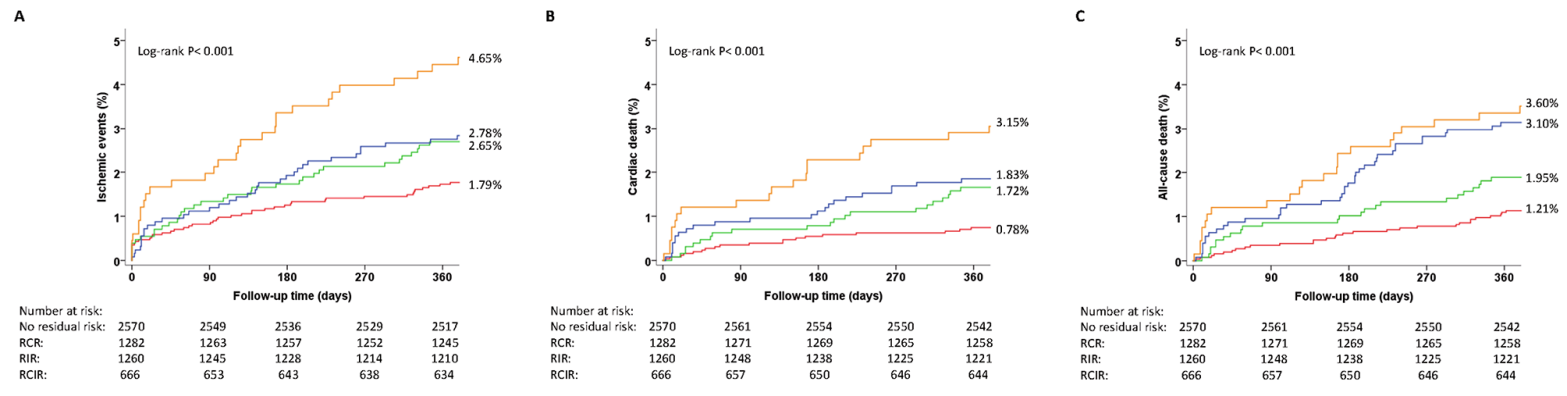
The cumulative Kaplan-Meier analyses according to residual risk defined by high-sensitivity C-reactive protein and remnant cholesterol (**A**. ischemic events **B**. cardiac death **C**. all-cause death) Abbreviations: RCR, residual cholesterol risk; RIR, residual inflammation risk; RCIR, residual cholesterol and residual inflammation risk;

The primary outcome of 12-month ischemic events occurred in 46 (1.79%), 34 (2.65%), 35 (2.78%), and 31 (4.65%) patients in the no residual risk, RCR, RIR, and RCIR groups, respectively (log-rank *P* < 0.001). The incidences of 12-month all-cause death and cardiac death were also greater in the RCIR group. The Kaplan‒Meier analysis results are shown in Fig. 3. Compared with no residual risk, the aHR (95% CI) of ischemic events for RCR, RIR, and RCIR were 1.69 (1.08–2.66), 1.35 (0.86–2.13), and 2.80 (1.75–4.49), respectively; the aHR (95% CI) of cardiac death were 2.61 (1.41–4.84), 1.89 (1.02–3.49), and 4.10 (2.18–7.70), respectively; the aHR (95% CI) of all-cause death were 1.87 (1.10–3.20), 1.99 (1.23–3.23), and 3.00 (1.73–5.19), respectively (Table 4). The adjusted Kaplan‒Meier analysis results are shown in Supplementary 4.

**Table 4:** Cox regression analyses of residual risk for predicting clinical outcomes

	Incidence (%)	Hazard ratio (95%CI)	*P* value	Adjusted hazard ratio (95%CI)	*P* value
**Ischemic events**					
No residual risk	1.79% (46/2570)	Reference	-	Reference	-
RCR	2.65% (34/1282)	1.49 (0.96–2.32)	0.0785	1.69 (1.08–2.66)	0.0224
RIR	2.78% (35/1260)	1.56 (1.01–2.43)	0.046	1.35 (0.86–2.13)	0.1881
RCIR	4.65% (31/666)	2.64 (1.67–4.16)	< 0.0001	2.80 (1.75–4.49)	< 0.0001
**Cardiac death**					
No residual risk	0.78% (20/2570)	Reference	-	Reference	-
RCR	1.72% (22/1282)	2.21 (1.21–4.06)	0.0101	2.61 (1.41–4.84)	0.0023
RIR	1.83% (23/1260)	2.37 (1.30–4.31)	0.0048	1.89 (1.02–3.49)	0.0421
RCIR	3.15% (21/666)	4.11 (2.23–7.58)	< 0.0001	4.10 (2.18–7.70)	< 0.0001
**Death from any cause**					
No residual risk	1.21% (31/2570)	Reference	-	Reference	-
RCR	1.95% (25/1282)	1.62 (0.96–2.75)	0.0714	1.87 (1.10–3.20)	0.0216
RIR	3.10% (39/1260)	2.59 (1.62–4.16)	0.0001	1.99 (1.23–3.23)	0.0053
RCIR	3.60% (24/666)	3.03 (1.78–5.17)	< 0.0001	3.00 (1.73–5.19)	< 0.0001

Model adjusted for age, sex, hypertension, diabetes, previous myocardial infarction, previous percutaneous coronary intervention, previous stroke, smoking, type of ACS, anemia, eGFR, arterial access, coronary arteries treated, and number of stents

Abbreviations: RCR, residual cholesterol risk; RIR, residual inflammation risk; RCIR, residual cholesterol and residual inflammation risk

### Subgroup analysis

**Fig. 4:**
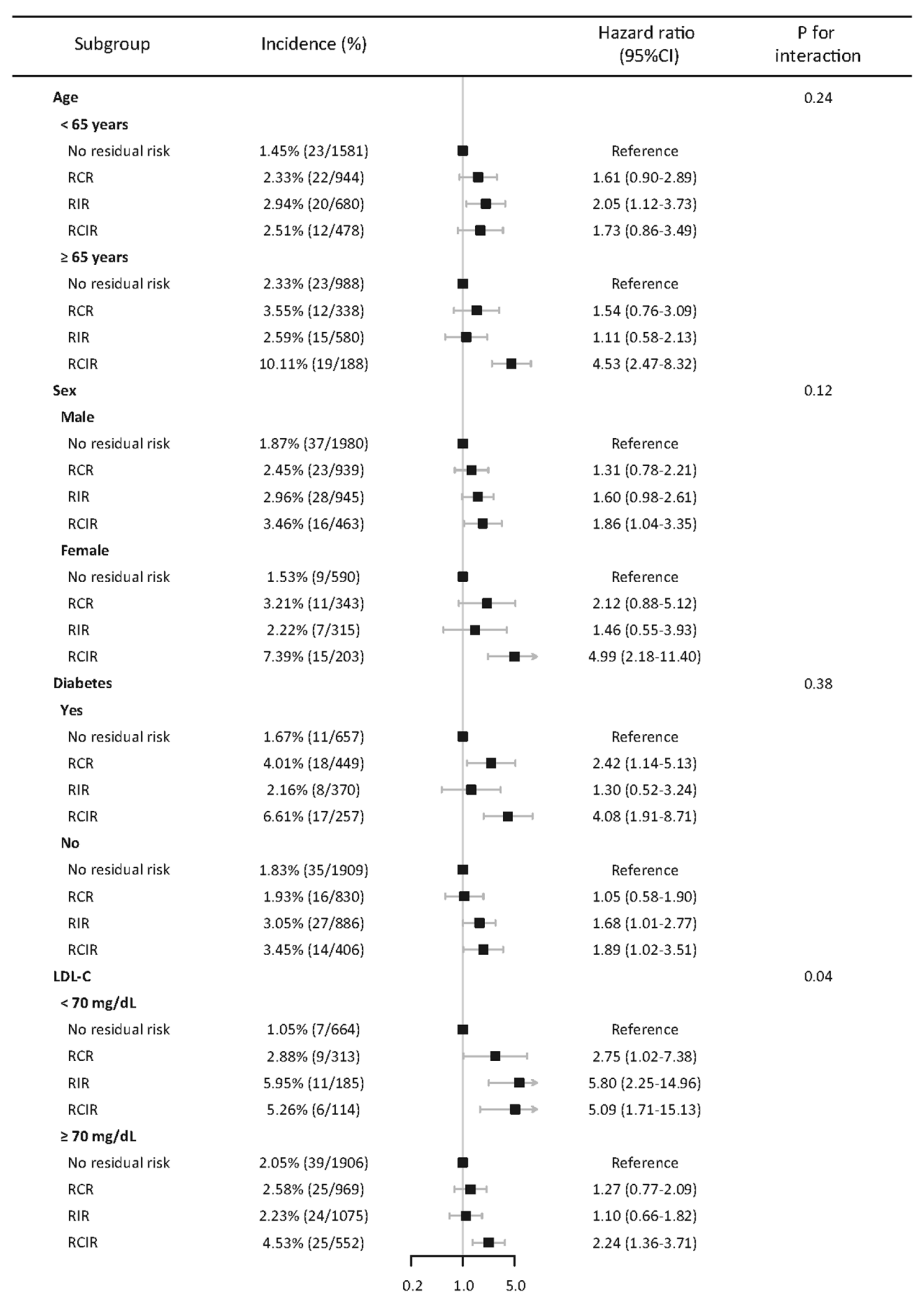
Forest plot of subgroup analysis for ischemic events according to residual risk defined by high-sensitivity C-reactive protein and remnant cholesterol Abbreviations: RCR, residual cholesterol risk; RIR, residual inflammation risk; RCIR, residual cholesterol and residual inflammation risk; LDL-C, low-density lipoprotein cholesterol

The subgroup analysis results for the primary outcome of ischemic events at 12 months are shown in Fig. 4. No statistically significant interactions were detected between age (< 65 years vs. ≥65 years), sex (male vs. female), or diabetes status (yes vs. no) (all *P*_*interaction*_ > 0.05). However, a significant difference was found in patients with and without LDL-C < 70 mg/dL (*P*_*interaction*_ = 0.04). In patients with adequate LDL-C control (< 70 mg/dL), compared with no residual risk, the HR (95% CI) of ischemic events for RCR, RIR, and RCIR were 2.75 (1.02–7.38), 5.80 (2.25–14.96), and 5.09 (1.71–15.13), respectively. In patients with elevated LDL-C levels (≥ 70 mg/dL), the corresponding values were 1.27 (0.77–2.09), 1.10 (0.66–1.82), and 2.24 (1.36–3.71), respectively.

### Additional effects after adding hsCRP and RC to GRACE score

As shown in Table 5, adding hsCRP or RC separately to the GRACE score independently improved the predictive value for both the primary and secondary outcomes. Furthermore, the predictive performance of the GRACE score model was further enhanced when both hsCRP and RC were incorporated into the model [AUC: ischemic events: 0.64 (95% CI, 0.60–0.69) vs. 0.66 (95% CI, 0.62–0.71), *P* = 0.003; cardiac death: 0.70 (95% CI, 0.64–0.76) vs. 0.74 (95% CI, 0.68–0.79), *P* < 0.001; all-cause death: 0.71 (95% CI, 0.66–0.76) vs. 0.73 (95% CI, 0.69–0.78), *P* < 0.001].

**Table 5:** Evaluation of predictive models for clinical outcomes

Outcomes	AUC (95%CI)	*P* value
**Ischemic events**		
GRACE score	0.64 (0.60–0.69)	-
GRACE score + hsCRP	0.65 (0.60–0.69)	0.032
GRACE score + RC	0.65 (0.61–0.70)	0.039
GRACE score + hsCRP + RC	0.66 (0.62–0.71)	0.003
**Cardiac death**		
GRACE score	0.70 (0.64–0.76)	-
GRACE score + hsCRP	0.72 (0.66–0.77)	0.009
GRACE score + RC	0.72 (0.67–0.78)	0.013
GRACE score + hsCRP + RC	0.74 (0.68–0.79)	< 0.001
**Death from any cause**		
GRACE score	0.71 (0.66–0.76)	-
GRACE score + hsCRP	0.72 (0.68–0.77)	0.004
GRACE score + RC	0.72 (0.67–0.77)	0.074
GRACE score + hsCRP + RC	0.73 (0.69–0.78)	< 0.001

## Discussion

The present study, utilizing data from a large real-world registry, aimed to assess the potential of combining hsCRP with RC for improving risk stratification for ACS patients on contemporary statin treatment undergoing PCI. The main conclusions are as follows: (1) elevated hsCRP and RC levels were both independently associated with a greater risk of ischemic events, cardiac death and all-cause death after controlling for potential confounders; (2) patients with simultaneous risks of residual inflammation and residual cholesterol had a greater risk of ischemic events, cardiac death and all-cause death, especially in patients with adequate LDL-C control (LDL-C < 70 mg/dl); and (3) adding hsCRP and RC to GRACE score models can significantly enhance the ability to predict adverse clinical outcomes.

Mechanistically, RC consists of triglyceride-risk lipoproteins that generally do not penetrate the arterial wall due to their larger particle size compared with the fenestra’s size in the elastic lamina of the media [25]. However, these particles can also slowly enter the intima and contribute significantly to the development and progression of atherosclerosis because they contain more cholesterol per particle and are more prone than LDL particles to be taken up by macrophages in the arterial wall [25, 26]. A Mendelian randomization study indicated that regardless of LDL-C or HDL-C levels, a 2.8-fold increase in the causative risk of ischemic heart disease was linked to every 1.0 mmol/L increase in RC [27]. Inflammation is also crucial for the development and subsequent rupture of arterial plaques, which can lead to atherosclerotic cardiovascular disease and potentially trigger an MI [28]. From the CANTOS trial to LoDoCo2 trial, anti-inflammatory medications that target certain inflammatory pathways demonstrated the potential to lower the risk of cardiovascular events in patients with chronic coronary disease and those who have experienced MI [29–31]. Given the negative interactions between inflammation and lipids in several processes associated with plaque formation and rupture, it is physiologically feasible that simultaneous increased levels of RC and hsCRP confer the highest risk of ischemic events, cardiac death, or all-cause death, as demonstrated in the present study.

Regarding the relative effects between the RIR and RCR, a previously published collaborative analysis revealed that inflammation assessed by hsCRP or IL-6 serves as a more robust predictor of future cardiovascular events and all-cause mortality than cholesterol evaluated as LDL-C [7, 8]. Nevertheless, in ACS patients on statin treatment undergoing PCI, the findings indicate that the substitution of LDL-C with RC in assessing RCR appears to possess at least a comparable level of predictive capability for the risk of future ischemic events compared with the RIR evaluated by hsCRP. This result may be attributed to the unique physicochemical properties of remnant particles. First, as mentioned before, these remnant particles have a similar atherogenic propensity to that of LDL particles but contain approximately 40 times more cholesterol [32]. Second, unlike LDL, remnants may not need to undergo oxidation before being digested by macrophages, leading to foam cell formation and subsequent inflammation that contributes to the development of atherosclerosis [25, 33, 34]. Moreover, prior investigations have also demonstrated that elevated blood levels of RC may more accurately indicate residual cardiovascular risk than LDL-C levels in primary [14, 16] and secondary prevention populations [17, 18].

Reducing LDL-C levels has been the main emphasis of current consensus and guidelines related to lowering the risk of cardiovascular events [35, 36]. Nevertheless, a significant percentage of patients continue to pose a high residual risk even after meeting treatment goals suggested by guidelines [3]. According to the subgroup analysis, the results revealed that individuals with adequate LDL-C control may face a 2-fold greater risk of ischemic events due to an increased dual residual risk, as evaluated through hsCRP and RC, than individuals whose LDL-C values are greater than 70 mg/dL. These findings appear to be consistent with prior research results indicating a correlation between RC and the overall amount of coronary atherosclerotic plaque burden in individuals with ideal LDL-C levels [37]. Another collaborative analysis of 10 trials has also revealed that increased on-treatment RC levels were substantially associated with a greater cumulative incidence of cardiovascular events among patients with robust reductions in LDL-C levels [38]. Therefore, even after LDL-C has been decreased to the recommended concentration in patients on lipid-lowering medication, the combined residual risk assessed by hsCRP and RC may be taken into consideration for directing additional treatment intensification [39].

Since RC and hsCRP are actively implicated in the formation of atherosclerosis beyond the influence of LDL-C, treatment with RC-lowering and anti-inflammatory drugs for minimizing the risk of cardiovascular diseases has previously been explored. According to recent research, individuals treated with proprotein convertase subtilisin/kexin type 9 inhibitors exhibited benefits with respect to decreased cholesterol remnants and lipid residual risk, beyond the reductions in LDL-C [40]. Specifically, a combined analysis of three randomized controlled trials suggested that alirocumab therapy resulted in a 42.1–52.5% decrease in RC levels compared with placebo [41]. Regarding anti-inflammatory therapies, the administration of canakinumab, an anti-interleukin-1β antibody, has demonstrated efficacy in decreasing the risk of recurrent cardiovascular events, independent of reduced lipid levels [29]. Moreover, large-scale randomized controlled studies have indicated that colchicine not only decreases ischemic events by 23% in recent MI patients but also reduces cardiovascular event risk by one-third among individuals with chronic coronary heart disease receiving standard therapy [30, 31]. Thus, with the continuous refinement of the concepts of RIR and RCR, as well as the ongoing development of targeted therapies, personalized cardiovascular care can be advanced to connect the most appropriate intervention measures with the most suitable patients to achieve precision medicine.

### Study strengths and limitations

To the best of our knowledge, this is the first study to utilize RC as an indicator of RCR in conjunction with hsCRP, a marker of RIR, to evaluate the combined impact of elevated dual risks on the prognosis of ACS patients receiving statin treatment who underwent PCI within a large and contemporary cohort derived from real-world data. Meanwhile, all clinical events were systematically monitored through a standardized assessment conducted by trained personnel at predetermined intervals, complemented by committee oversight, thereby bolstering the validity and reliability of the study outcomes. However, certain limitations should also be noted. First, this was a post hoc analysis of a sizable prospective single-center cohort of ACS patients who underwent PCI, which may impact the generalizability of the results. These findings require confirmation through more specifically designed studies. Second, the concentration of RC was computed instead of being measured directly. But the two methods have demonstrated good correlation [42], and the equation utilized has been shown to have independent prognostic value in multiple previous studies [15, 16, 43–45]. Third, the circulating lipid profile and hsCRP were measured subsequent to the procedure, which may not reflect the average levels during follow-up. The on-treatment data may have more clinical significance. Finally, a follow-up of 12 months post-discharge is recognized as relatively short. Thus, the impact of elevated dual risk on the long-term prognosis of patients requires further investigation.

## Conclusion

In summary, the current investigation suggested that elevated dual residual risks evaluated by hsCRP and RC are associated with adverse clinical outcomes in patients admitted for ACS who are receiving contemporary statin therapy and undergoing PCI. These novel findings suggested that, beyond LDL-C targeting, a comprehensive assessment of other residual inflammatory and cholesterol risk factors is necessary for enhanced risk stratification, which is crucial for improved clinical decision-making and patient management in ACS patients post-PCI.

### Electronic supplementary material

Below is the link to the electronic supplementary material.

Supplementary Material 1

## Data Availability

No datasets were generated or analysed during the current study.

## Abbreviations

ACS: Acute coronary syndrome
hsCRP: High-sensitivity C-reactive protein
RC: Remnant cholesterol
RIR: Residual inflammation risk
RCR: Residual cholesterol risk
RCIR: Residual cholesterol and residual inflammation risk
PCI: Percutaneous coronary intervention
LDL-C: Low-density lipoprotein cholesterol
HDL-C: High-density lipoprotein cholesterol
TC: Total cholesterol
