# Supplementary material for: The residual risk of inflammation and remnant cholesterol in acute coronary syndrome patients on statin treatment undergoing percutaneous coronary intervention
Source: Lipids Health Dis. 2024 Jun 7;23:172. doi: 10.1186/s12944-024-02156-3 (PMC11157837; doi:10.1186/s12944-024-02156-3)
Supplement: Supplementary file 1 — Supplementary Material 1 [file 12944_2024_2156_MOESM1_ESM.docx]

**Supplementary 1 The adjusted cumulative Kaplan-Meier analyses according to high-sensitivity C-reactive protein or remnant cholesterol**


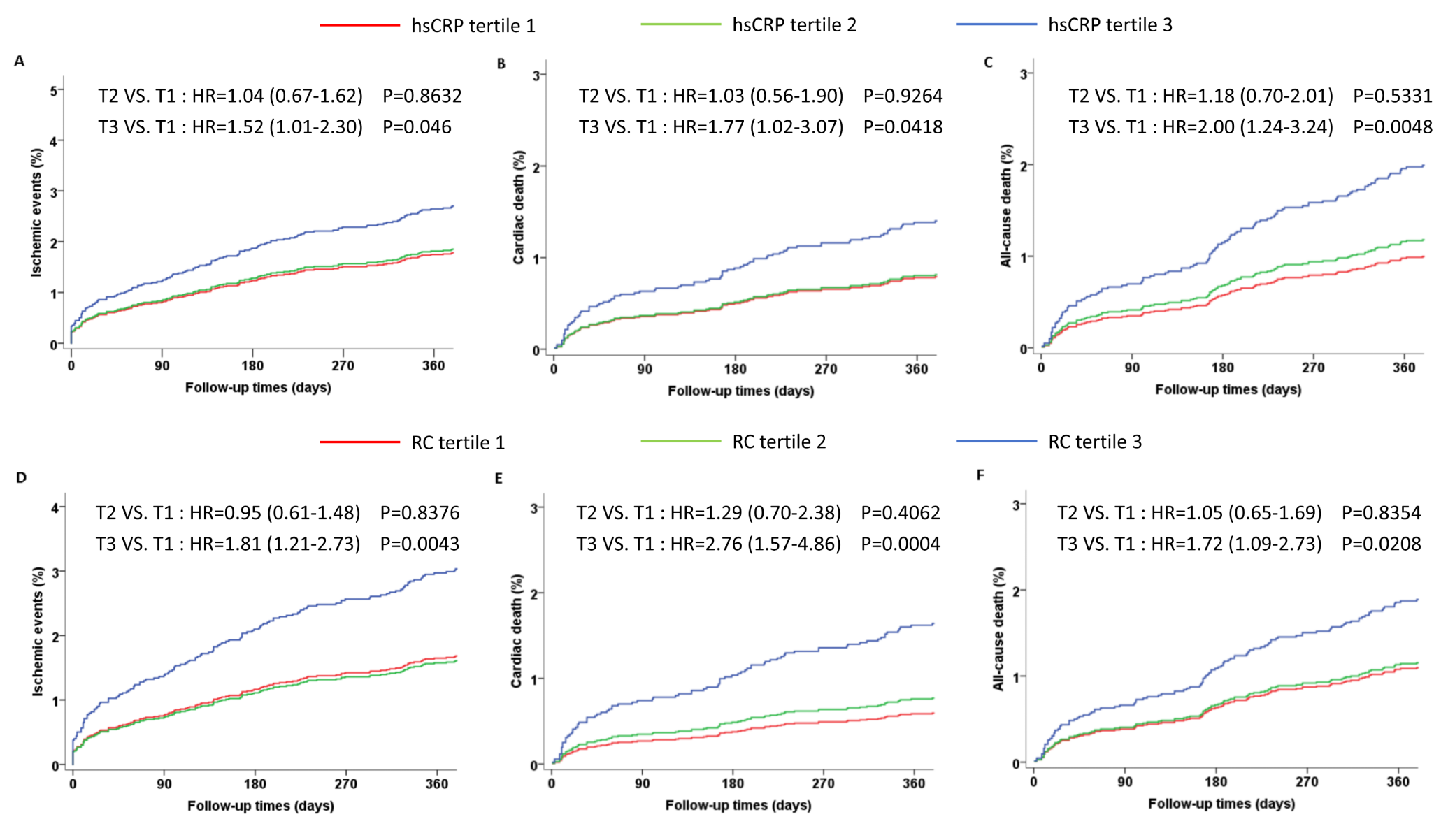


**(hsCRP: A. ischemic events B. cardiac death C.all-cause death; RC: D. ischemic events E.cardiac death F.all-cause death )**

Model adjusted for age, gender, hypertension, diabetes, previous myocardial infarction, previous percutaneous coronary intervention, previous stroke, smoking,

type of ACS, anemia, eGFR, arterial access, coronary arteries treated, and number of stents.

**Supplementary 2 Restricted cubic spline fitting for the association between high-sensitivity C-reactive protein and remnant cholesterol with cardiac death**


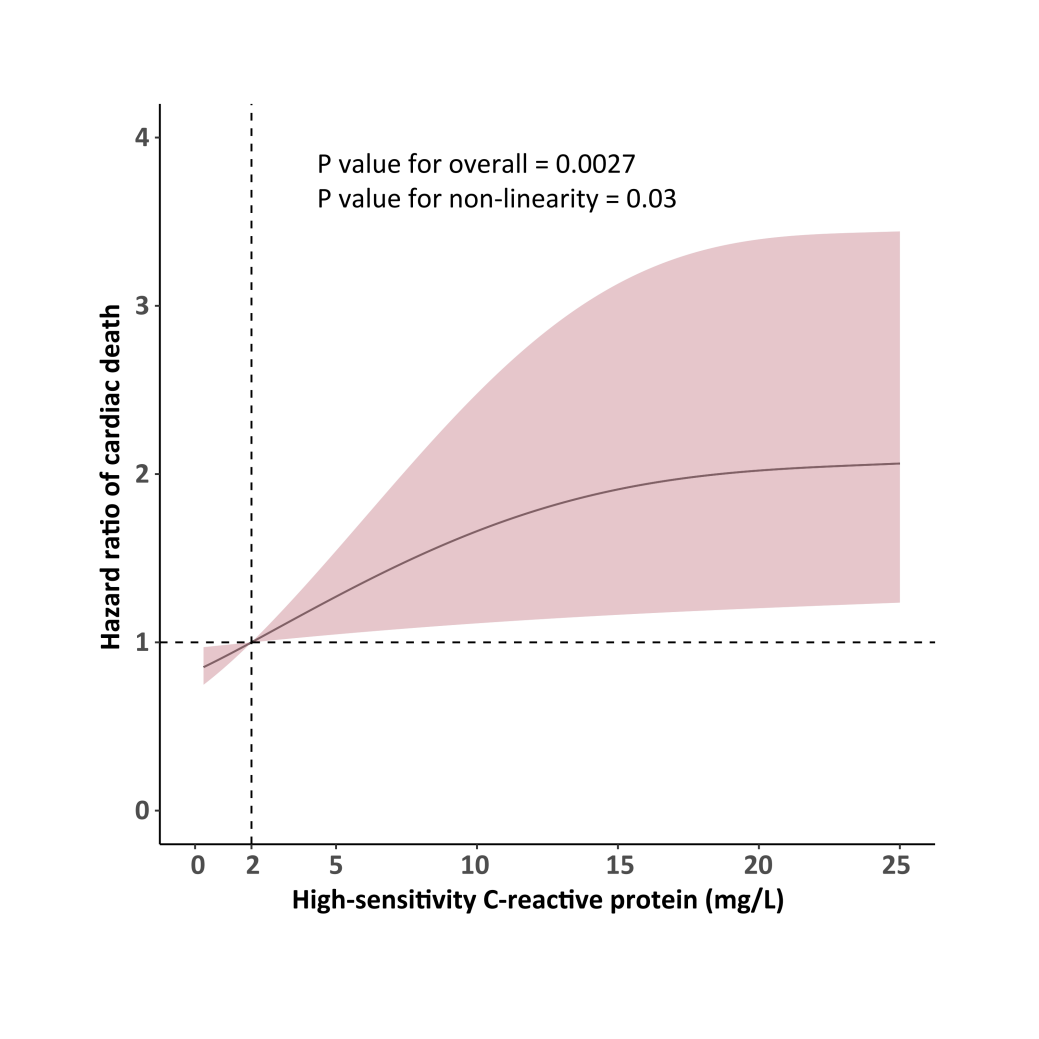

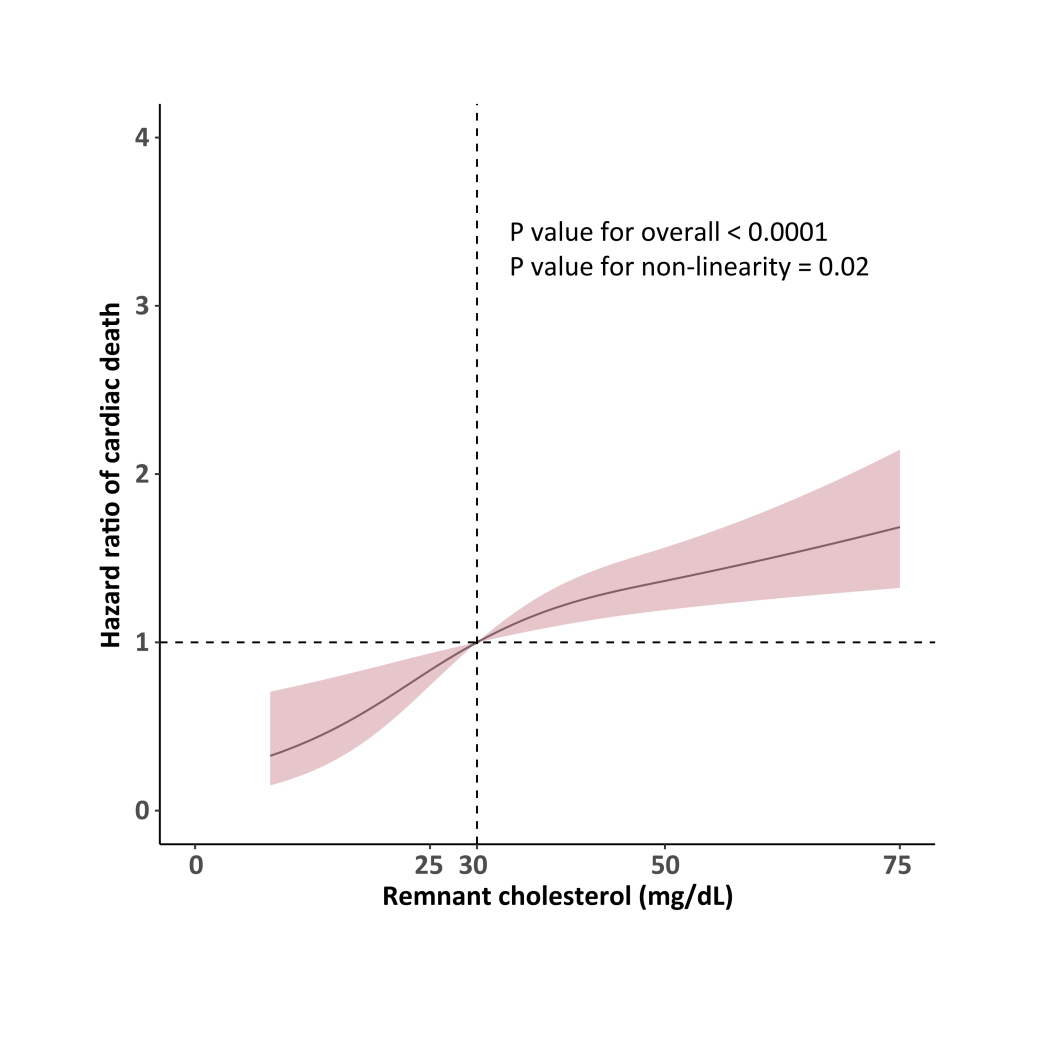


**Supplementary 3 Restricted cubic spline fitting for the association between high-sensitivity C-reactive protein and remnant cholesterol with all-cause death**


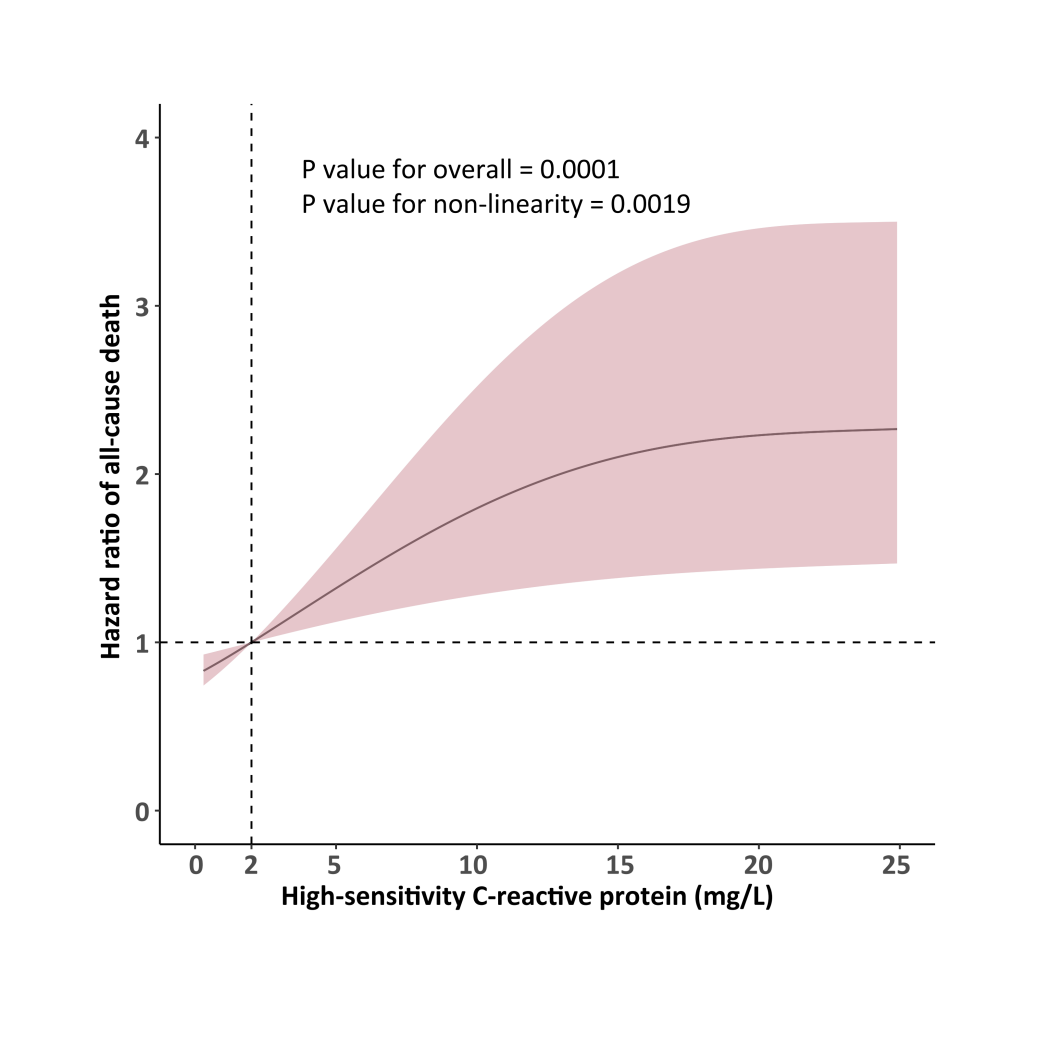

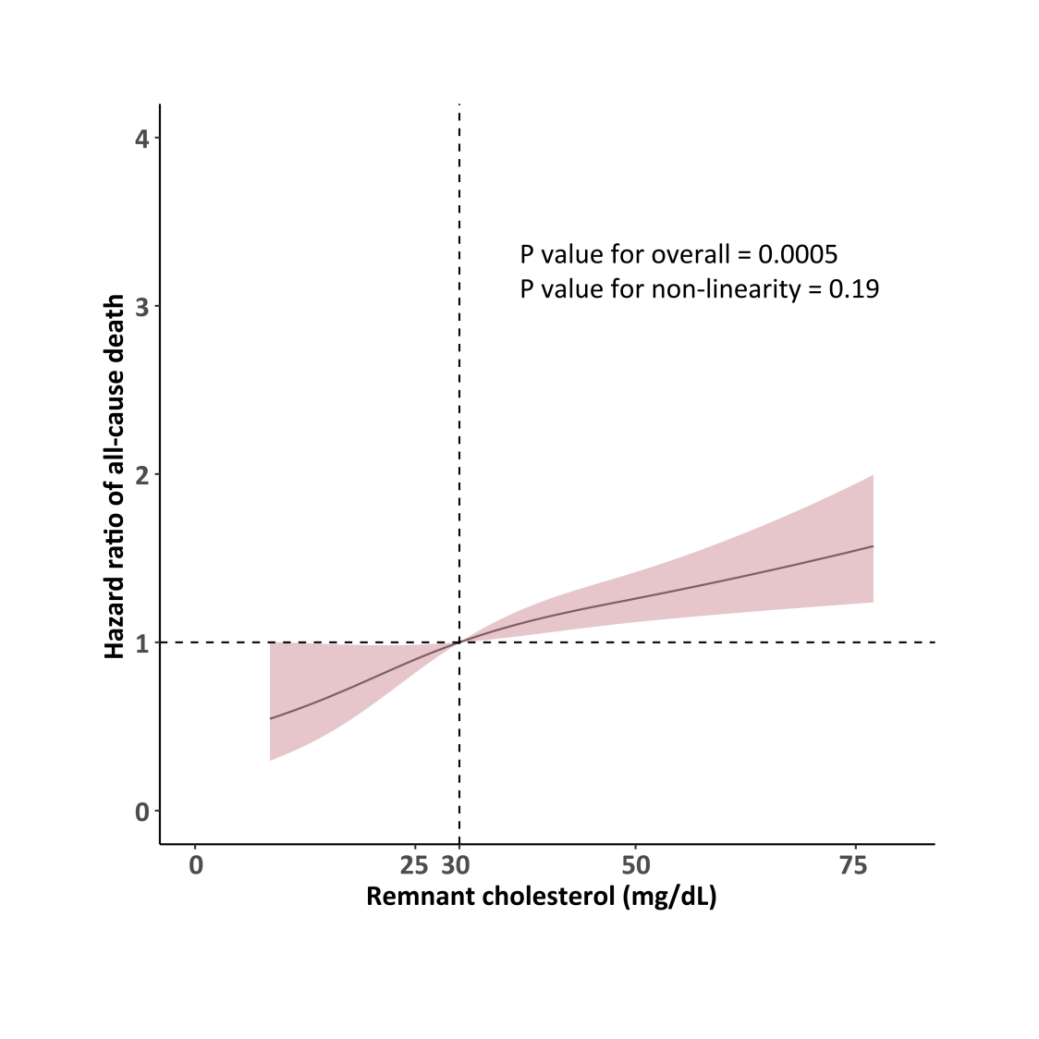


**Supplementary 4 The adjusted cumulative Kaplan-Meier analyses according to residual risk defined by high-sensitivity C-reactive protein and remnant cholesterol**


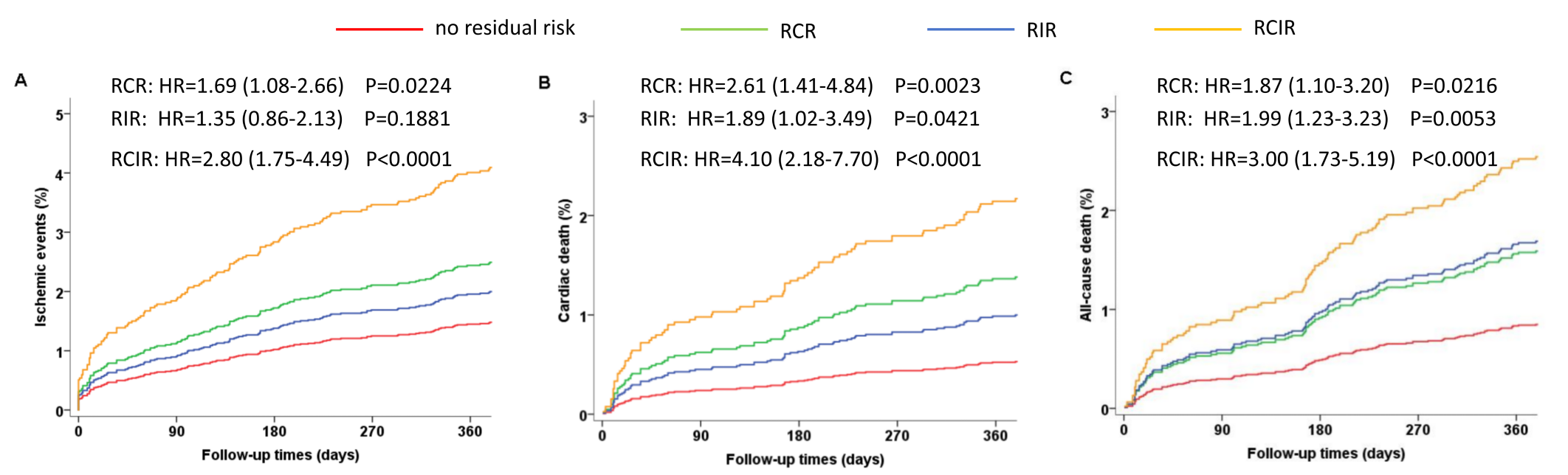


**(A. ischemic events B.all-cause death C.cardiac death)**

patients in hsCRP tertiles 1, 2 and RC tertiles 1,2 were defined with no residual risk; patients in hsCRP tertiles 1, 2 and RC tertiles 3 were defined with RCR; patients in hsCRP tertiles 3 and RC tertiles 1,2 were defined with RIR; and patients in hsCRP tertiles 3 and RC tertiles 3 were defined with residual cholesterol and residual inflammation risk (RCIR).

Abbreviations: RCR,residual cholesterol risk; RIR,residual inflammation risk; RCIR,residual cholesterol and residual inflammation risk;

Model adjusted for age, gender, hypertension, diabetes, previous myocardial infarction, previous percutaneous coronary intervention, previous stroke, smoking,

type of ACS, anemia, eGFR, arterial access, coronary arteries treated, and number of stents.
